# Puerperal Insanity

**Published:** 1903-12

**Authors:** J. W. Palmer

**Affiliations:** Alley, Ga.


### PUERPERAL INSANITY.*

By J. W. PALMER, M.D.,

ALLEY, GA.

Puerperal insanity is a generic term used to include all cases of mental derangement incident to pregnancy and its sequelæ.

I shall not dwell on the symptoms and differential diagnosis of mania and melancholia, but on the relations of insanity mostly incident to pregnancy and its sequelæ. It is too lengthy and tiresome to cover that ground here, when we can find an exhaustive description of it in any standard work on insanity.

* Read before the Georgia Medical Association, April, 1903.

Puerperal insanity is conveniently divided into three classes:

First, insanity of pregnancy.

Second, puerperal insanity proper.

Third, insanity of lactation.

The form of puerperal insanity may be mania or melancholia, and sometimes these forms end or pass into dementia. Mania is most always the form of insanity that occurs in puerperal insanity proper. Melancholia is the form generally met with in the first and third classes, and if dementia occurs it is usually in these classes. Mania occurs more frequently than the others. The per cent. of different classes are, insanity of utero-gestation 20 per cent., puerperal insanity proper 50 per cent., and insanity of lactation 30 per cent.

Extensive collected statistics from various institutions devoted to the treatment of insanity claim that 7.1 per cent. of all cases of insanity of females are due to puerperal origin, and that the disease develops during the child-bearing process. As to the frequency of puerperal insanity in relation to the number of cases confined, will say that my experience in the lying-in hospitals and asylums while in Baltimore and since I have been practicing medicine proves that we seldom meet with a case of puerperal insanity. Summing up the tabulated cases from the best authors and writers and from the present statistics, it is sufficient to say that one woman out of every four hundred who is confined becomes insane.

There is a form of insanity not considered in the other classes known as *transient mania during delivery*. This is a peculiar form of mental derangement sometimes observed during labor, and called temporary insanity. It is more accurately described as a kind of acute delirium produced in the latter stage of labor by the intensity of the suffering caused by pains. It is most apt to occur as the head is passing through the *os uteri*, or at a late period during the expulsion of the child. It may consist of merely a loss of control over the mind, during which the patient, unless carefully watched, might in her agony seriously injure herself or her child. Sometimes it produces actual hallucinations. This kind of mania, if it may be so called, is merely transitory in its character, and disappears as soon as the labor is over. From a medico-legal point of view it may be of importance, as it has been held by some that in certain cases of infanticide the mother has destroyed the child when in this state of transient frenzy and when she was irresponsible for her acts. In the treatment of this variety of delirium we must, of course, try to lessen the intensity of the suffering, and it is in such cases that administration of chloroform is justifiable and necessary. After this temporary insanity passes off, patient should be kept quiet for some time.

*Class 1.*—Insanity of pregnancy, which is, without doubt, the least of all three forms. The intense mental depression which, in many women accompanies pregnancy, and causes the patient to take a despondent view of the condition and to look forward to the result of her labor with the most gloomy apprehension, seems to be often only a lesser degree of the actual mental derangement which is occasionally met. In many the attack of insanity can be traced as developing itself out of the ordinary hypochondriasis of pregnancy. A larger proportion of cases occur in primiparæ than in multiparæ. The period of pregnancy at which mental derangement shows itself varies. Most generally it is at the end of the third month, or the beginning of the fourth month. We should not confound aggravated hypochondriasis with insanity of pregnancy, as the former usually lessens after the third month, while the latter only begins after that date.

Sometimes there is a tendency to dipsomania in the early months, which may be an exaggeration of the depraved appetite or morbid cravings so commonly observed in pregnant women, just as the melancholia may be a further development of lowness of spirit.

*Prognosis.*—The prognosis may be said to be on the whole favorable, though there is little hope of a cure until after the termination of pregnancy.

*Causes.*—By far the most prolific cause of insanity of pregnancy is an hereditary predisposition to insanity that exists, and the events of pregnancy acting simply as the spark that fires the mind. The morbid dread of pregnancy aids in its causation. Anemia, which comes on gradually, has an effect in producing it. The most recent theories show and claim that toxemia of pregnancy plays an important part, and claim that the toxemia is caused by toxins produced in bodies of mother and child, and retained because of a lack of excretion or by incomplete metabolism, or by both. These toxins may be the result of chemical decomposition of food and of the waste material of tissue change largely increased by pregnancy. The insanity may be caused by a yet unknown group of toxins which may exist in the body during the period of gestation.

*Class 2.*—Puerperal insanity *proper.* True puerperal insanity has always attracted much attention from obstetricians, often to the exclusion of other forms of mental derangement connected with the puerperal state. Puerperal insanity proper is the most interesting part of the subject. We may define it to be that form of insanity which comes on within a limited period after delivery, and which is intimately connected with that process, because the mental disturbance, which is usually acute confusional insanity, is due to toxemic infection. Mania is generally the form of insanity met with, but by no means is it the only kind of insanity observed, for there is a considerable number of well-marked examples of melancholia. My experience and the authority of the best writers justify me in saying that all cases of acute mania come on within eight days after delivery, and that all cases of melancholia develop themselves after that period.

It is pretty generally admitted by all alienist physicians that hereditary tendencies form one of the strongest predisposing causes of mental disturbance in the puerperal state. In a large proportion of cases circumstances producing debility and exhaustion or mental depression have preceded the attack, preceded by post-partum hemorrhage, severe and complicated labor, or they may have been weakened by over-frequent pregnancies, or by lactation during the early months of pregnancy.

Morbid dread during pregnancy, insufficient to produce insanity before delivery, may develop into puerperal insanity after labor. Shame and fear of exposure in unmarried women not infrequently lead to the disease. The anemia produced by post-partum hemorrhage is a potent factor in producing puerperal insanity. Mental impressions may be the determining cause in predisposed persons. The age of the patient has some influence, and there seems to be a greater liability at advanced ages, especially when such women are pregnant for the first time. The symptoms are intensified by disturbance of the abdominal or pelvic organs. The most recent theories and investigations attribute the causation of the disease to some morbid condition of the blood. The possibility and probability of the acute form of puerperal insanity, coming on shortly after delivery, being dependent on some form of septicemia is one that deserves most careful consideration and investigation on this subject. There are several theories advanced as to the dependence of the disease being due to some morbid condition of the blood. Some advance and advocate the uremic blood-poisoning theory, while others claim it is due to hepatic insufficiency.

Puerperal infection or toxemic infection is the direct cause of puerperal insanity, based on the following reasons: That puerperal insanity occurs in the great majority of cases within the first ten days after delivery, the same period during which puerperal infection usually occurs. It is generally accompanied by elevation of temperature and other evidences of febrile disease. The clinical form in which puerperal insanity manifests itself is in most cases that of acute delirious or confusional mania, closely resembling febrile delirium. The death-rate is much higher than in simple mania, death occurring from exhaustion, usually high temperature and rapid pulse.

Post-mortem examinations though apparently infrequent in these cases, have shown grave involvement of the pelvic organs. Examinations of the pelvic organs during life show lacerations of the perineum and cervex uteri, which are the channels of infection in the puerperal woman. Sometimes there are secondary conditions found, such as intrapelvic inflammations and consequent abnormal locations, fixations and congestion of the uterus, tubes and ovaries. It should be borne in mind that in each individual case many factors are indissolubly associated, the patient's mental breakdown being the result of several complex conditions, each reacting upon and intensifying the other. I think that the renal and hepatic theories are fallacious, but do believe they have some weight in producing the disease, because effect of faulty elimination of secretion altered in quality and quantity plays an important role in the production of the unstable nervous system of pregnant women, so we find incapacity of liver to perform the work thrust upon it and failure of skin and kidneys, all of which certainly can and do induce faulty nutrition of the brain. There are many facts, as statistics of recent years show, to support the belief that I have advocated, that sepsis is by far the most important cause of puerperal forms of insanity. But it is strange that no one has as yet ventured to assert that all puerperal cases are due to intoxication from either bacteria or toxemic organic compound. In short, I venture to say that *the hereditary taint and morbid predisposition are the true causes of the disease, the puerperal condition being its immediate and exciting cause.* Recent advances in bacteriology warrant the belief that some day proof will be abundant of the universal belief that either toxemic or septic infection is a primary factor in all psychoses of childbirth. In no class of cases are gynecological investigations of more importance than in the study of the causation of puerperal insanity.

*Diagnosis.*—The diagnosis of puerperal insanity can not be made from the symptoms. It is not a special variety of insanity symptomatically, but etiologically.

*Prognosis.*—The prognosis is favorable; 75 to 80 per cent. recover, but a larger proportion die of exhaustion.

In no class of cases of insanity is the prognosis so favorable as those of puerperal origin. The disease may resolve itself into a consideration of immediate risk to life and of the chances of ultimate restoration of the mental faculties. It is an old aphorism "that mania is more dangerous to life and melancholia to reason." Extreme rapidity of the pulse is indicative of a fatal tendency. If pulse 120, conditions grave, but as long as pulse remains below 100 no immediate danger. Should there be development of phlegmasia dolens, the prognosis is exceedingly grave. The most dangerous class of cases are those attended with some imflammatory complication, with marked elevation of temperature.

*Duration.*—The duration of the disease varies considerably, generally getting well inside of three months, but melancholia generally lasts longer than mania.

*Class 3.*—Lactational insanity. It is a mental disturbance occurring during the period of lactation, usually coming on from six weeks to ten months after labor; the form of insanity is generally melancholia, and occasionally you meet with confusional insanity, which is much more transient than in true puerperal insanity. There seems, however, to be more risk of the insanity becoming permanent than in other forms. Sometimes the melancholia degenerates into dementia and the patient becomes hopelessly insane.

*Etiology.*—Hereditary taint is an important factor in producing this form of insanity, as in the other forms. Prolonged or excessive lactation is considered the chief cause in producing lactational insanity. Careful inquiry, however, shows that certain conditions favoring toxemic infection are often present, as a mammitis or mammary abscess sometimes precedes the mental disturbance. Defective uterine involution is considered as a factor. Lacerated cervix and endometritis have been found present in some cases. Its dependence on causes producing anemia and exhaustion plays an important part. Retained placenta, I believe, has force in producing it, as in a case I treated with lactational insanity. I found her with discharge and placental retention six weeks after labor. The toxemic effects from this retained placenta in said case, giving rise to slight febrile condition, prove that toxemic conditions are important factors in producing lactational insanity.

*Diagnosis.*—The occurrence of confusional insanity during the nursing period is the only diagnostic feature. There is nothing distinctive in the symptomatology. Three or four per cent. of all cases of insanity in women occur during the nursing period.

*Prognosis.*—Prognosis is moderately favorable; from forty to fifty per cent. recover. The danger to life is not great if the cause producing debility be recognized and at once removed.

*Mania and Melancholia.*—The symptoms of these forms of insanity are practically the same as in the non-pregnant state; consequently I will dwell only on the symptoms peculiar and incident to puerperal insanity, for you can find very extensive description of the symptoms in any standard work on insanity. Mania, more frequently than melancholia, and occurring generally in puerperal insanity proper, almost always comes on in the first week of the lying-in period. It is almost always related to certain well-known symptoms of puerperal sepsis. Fever nearly always present. There may be prodromic symptoms. Changes in the quantity and character of the lochia are present. The outbreak usually begins with excitement, rapidly ending in incoherence. The usual feelings are perverted. The patient may have attacks of violence, during which attempts are made on the life of the husband, the newly-born babe or other children, if any.

These symptoms often have a religious basis; at other times they are based on delusions of jealousy. Usually the hallucinations and delusions of the patient are of a sexual character. The most refined women will surpass the imagination of the most degraded in their obscenity and vulgarity of language and actions. The eyes are bright, face pallid, pulse quick, tongue dry and furred, bowels mostly constipated, urine high colored and scanty and appetite uncertain. They will sometimes try to swallow anything, such as bedclothes, and will try to gouge out their own eyes. The patients often seem dissatisfied with their attending physician and nurses. They will often yield to suggestions and treatment of physician on first visit, though no longer, but beg for another physician. I experienced this in treating one case in which she became dissatisfied and absolutely refused to follow any of my directions, or take one dose of medicine from me. Another physician was called in; after his first visit he was treated likewise, and the third physician was called, and after his first visit he received like treatment. Motor excitement is common. Some desire to remove their clothing, in some probably a desire to expose the body to view, while in others it is probably due to hallucination of common sensation, rendering the weight or pressure of the clothing unbearable.

*Melancholia.*—This most commonly occurs in utero-gestation and lactation, and is often preceded by cephalalgia, tinnitus aurium and flashes of light before the eyes, and other indications of debility when it comes on during lactation. But when in utero-gestation, the accompanying delusions are generally exaggerations of the anxieties and whims of pregnancy. The advent of melancholia is more gradual. It may commence with depression of spirit without any adequate cause, associated with insomnia, disturbed digestion, headache, and other indications of bodily derangement. Such symptoms, showing themselves in women who have been nursing for any length of time, or in whom any other evident cause of exhaustion exists, should never pass unnoticed. Soon the signs of mental depression increase and positive delusions show themselves. In some cases, which approach in character those of mania, there is considerable excitement, rapid pulse and furred tongue and much restlessness. Acute cases of melancholia, coming on during puerperal state, most often assume this form. In others there are less of these symptoms; the patient sits for hours without speaking or moving; but there is not much excitement, and this is the form most characteristic of insanity of lactation. There is always a strong tendency to suicide, and it should never be forgotten in melancholic cases that it may develop itself in an instant and that a moment's carelessness on the part of the attendants may lead to disastrous results.

*Treatment.*—The indications for treatment are to check profuse, exhausting discharge, to support the patient's strength and to insure perfect quiet. With the first sign of trouble the child should be taken from mother's sight and breast, liquid food should be given at frequent intervals, care should be taken to keep the bladder and rectum empty, the room should be darkened and its temperature should be regulated. We must calm the excitement and give rest to the brain. In acute mania there are three important things to do: We must seek and remove the cause, bearing in mind that puerperal insanity is an infectious pychosis; the local source of infection should be sought out and removed, if possible. In some cases there is simple sapremia due to absorption of septic materials from the birth canal. In others there is septicemia. In short, will say treatment is the same as in puerperal fever without insanity. Streptococcic serum and intravenous infusion of formalin are used and advocated by some, but I have had no experience with them. Patient should have plenty of nourishment. If no febrile condition, give solid food as long as you can, but if any febrile condition, only give liquid food; but soon as possible give solid food, as it is much better. Give the liquid food frequently and in small quantities; if patient refuses to take food, give it to her forcibly. Alcoholic stimulants should be given to the fullest extent, when well borne.

At the onset a good purgation is beneficial; after that it is harmful, but the liver and bowels should be kept in good condition by giving mild laxatives. When diuretics are indicated, give potassium acetate and infusion digitalis. The heart should be watched and its strength maintained by small and repeated doses of digitalis, so as to keep the brain well nourished. Opiates should not be given unless when in severe pain, as in cellulitis, for it has adverse action on the secretions. To produce sleep and calm the patient, of all remedies, chloral hydrate stands first, and given with potassium bromide, its hypnotic action is increased. Trional is a very reliable drug for maniacal excitement and insomnia, given in doses of thirty grains every two to four hours to produce sleep and quiet patient, but must watch bowels and heart. I have found nothing to control the excitement better than hyoscin hydrobromate given by hypodermic in doses from 1-100 to 1-60, repeated once or twice daily, watching its effects. When patient convalesces should have a good tonic, change of air and scenery. There should be secured for patient rest, sleep, nutritious food and a daily evacuation of the bowels, and little by little she should be brought back to old habits and the responsibility of existence.

